# The Role of the Neuro-Astro-Vascular Unit in the Etiology of Ataxia Telangiectasia

**DOI:** 10.3389/fphar.2012.00157

**Published:** 2012-09-17

**Authors:** Leenoy Meshulam, Ronit Galron, Sivan Kanner, Maurizio De Pittà, Paolo Bonifazi, Miri Goldin, Dan Frenkel, Eshel Ben-Jacob, Ari Barzilai

**Affiliations:** ^1^School of Physics and Astronomy, Tel Aviv UniversityRamat Aviv, Israel; ^2^Sagol School of Neuroscience, Tel Aviv UniversityRamat Aviv, Israel; ^3^Department of Neurobiology, Faculty of Life Sciences, Tel Aviv UniversityRamat Aviv, Israel

**Keywords:** astrocyte, reactive gliosis, Ataxia Telangiectasia, DNA damage response

## Abstract

The growing recognition that brain pathologies do not affect neurons only but rather are, to a large extent, pathologies of glial cells as well as of the vasculature opens to new perspectives in our understanding of genetic disorders of the CNS. To validate the role of the neuron-glial-vascular unit in the etiology of genome instability disorders, we report about cell death and morphological aspects of neuroglia networks and the associated vasculature in a mouse model of *Ataxia Telangiectasia* (A-T), a human genetic disorder that induces severe motor impairment. We found that A-T-mutated protein deficiency was consistent with aberrant astrocytic morphology and alterations of the vasculature, often accompanied by reactive gliosis. Interestingly similar findings could also be reported in the case of other genetic disorders. These observations bolster the notion that astrocyte-specific pathologies, hampered vascularization and astrocyte-endothelium interactions in the CNS could play a crucial role in the etiology of genome instability brain disorders and could underlie neurodegeneration.

## Introduction

Over the past two decades a large body of evidence has emerged supporting the possibility that glia cells are not playing second fiddle to neuronal activity but rather they hold a key role in brain functionality. Astrocytes are the most abundant type of glial cells in the brain and are characterized by a complex morphology with highly degree of branching. They are involved in synaptic glutamate uptake, metabolic support of the neuronal cell bodies, and processes and contracts with blood vessels (Verkhratsky and Parpura, 2010; Barzilai, 2011). Under normal physiological conditions, astroglial cells buffer extracellular K^+^concentrations regulate neurotrophic factors release, and control extracellular concentrations of neurotransmitters released from neighboring synapses. In addition, astrocytes regulate water movement and distribution and are capable of neutralizing reactive oxygen species (ROS) in the vicinity of neurons and other types of cells. Astrocytes play a major role in metabolic support of adjacent neuronal cells by absorbing glucose from the nearby blood vessels, converting it to lactate, and providing it to neurons. Under pathological conditions, astrocytes experience metabolic stress and may contribute to CNS damage. Under these conditions astrocytes release ROS and NO, which in turn can generate highly toxic radicals such as hydroxyl radical and peroxynitrite. Pathological astrocytes fail to regulate water homeostasis, thereby favoring the insurgence of edema. Under these conditions, a reversal of neurotransmitter transporters take place and high levels of different substances are released together with cytotoxic levels of Ca^2+^ contributing to glutamate cytotoxicity. Failure to buffer extracellular K^+^ concentrations promotes further over-excitation of neuronal cells. Under these conditions glial cells secrete proinflammatory factors in conjunction with ROS, further exacerbating CNS damage.

Astrocytes are subdivided into two classes based on their morphological features and location in the CNS. Fibrous astrocytes are predominat in the gray matter, while in the white matter protoplasmic astrocytes are the majority (Miller and Raff, 1984). Few relevant features characterize human astrocytes in comparison to rodents, the most studied animal model. First, astrocytes in higher primates display a much larger complexity. Second, the human protoplasmic astrocytes have about 40 main processes with far more complex ramifications compared to mouse astrocytes. Moreover, every human protoplasmic astrocyte can enwrap about two million synapses, while only 100,000 synapses are covered by the processes of mouse astrocytes (Oberheim et al., 2006).

Astrocytes, apart “ruling” on their own territories, form functional networks, can display various shapes and carry out numerous functions. Although astrocytes express receptors and ion channels similarly to neurons, they do not produce action potentials but display calcium events which can propagate and synchronize with nearby cells. Furthermore, astrocytes act as pluripotent neural precursors supporting adult neurogenesis (reviewed in Verkhratsky and Steinhauser, 2000; Volterra and Meldolesi, 2005; Haydon and Carmignoto, 2006).

The potential importance of the astrocytic biochemical dynamics at single cell and network level has motivated parallel theoretical investigations aimed at unraveling the possible functional role of such astrocytic signaling. For example, intensive investigation was devoted to develop a biophysical model of glutamate-induced astrocytic intracellular calcium signals which could capture the essential biochemical features of the regulatory pathway of inositol 1,4,5-trisphosphate (IP3; De Pittà et al., 2009, 2011). While previous attempts to depict the complicated intracellular calcium dynamics did not provide a comprehensive biological description, this latter model focuses on the interplay between calcium dynamics and astrocyte functionality brought forth by the regulation of the intracellular second messenger IP_3_. At network level, one of the most interesting features of astrocytic calcium dynamics is their capability to communicate with each other over long distances by calcium waves. Such waves spread intercellularly through molecular gates called gap junctions, which connect directly intracellular spaces of each of the cells in the network. Computer modeling aimed at discovering what biophysical mechanisms could support long-distance propagation of calcium wave signaling suggests that IP_3_ diffusion through gap junctions is non-linear (Goldberg et al., 2010). This is a rather unexpected prediction since gap junctions are classically considered to allow linear (Fickian) diffusion. Along with necessary experimental verification, these new ideas provide another avenue in the process of deciphering long-distance communication between astrocytes.

## Astrocytes in Brain Dysfunction

Recent evidences strengthen the doctrine that glial cells play a crucial role in both neural functionality and brain activity. These findings should lead us to reconsider their contribution in neuropathology and injury as well (Seifert et al., 2006). This global perspective in the normal brain might yield new insights about how glial cells may participate in the pathogenesis of common neurological disorders such as Alzheimer’s and Parkinson’s diseases, stroke, epilepsy, and primary brain tumors (Bachoo et al., 2004). As a result, the investigation of brain disorders becomes a complex multi-aspect problem, where neurons and glial cells are equally influential as they are closely reacting with each other.

Damage to, or injury in the CNS results in the formation of a “glial scar” (Fawcett and Asher, 1999). This is consistent with reactive gliosis which occurs in order to facilitate the containment of the injury and the initiation of repair processes. It includes microglia recruitment, higher presence of oligodendrocyte precursors, mobilization of meningeal cells and a combined morphological-functional alteration of the astrocytes. Because astrocytes constitute nearly half of the cells in the human brain, there is essentially no CNS disease that does not involve them. That is, following a traumatic tissue injury, astrocytes undergo significant morphological changes and thereby functional alterations, which are usually referred to as reactive gliosis (astrogliosis) state. In response to the CNS damage, astrocytic structural changes are accompanied by the secretion of regulatory molecules which astrocytes express such as VEGF, endothelins, and ephrins/Eph-receptors (Li et al., 2007). These array of different molecules are known to modulate neuronal development, axonal growth, and vasculogenesis (Fagan et al., 2004; Eichmann et al., 2005). The astrocyte reactive response was found to be substantially involved in most brain dysfunctions. Remarkably, astrocytes are capable of accurately adjusting their reaction to the specific insult, according to the relevant characteristics of the pathology, the site of injury (Ridet et al., 1997), the stage of development, and the magnitude of the damage. They determine the type and the quantity (Eddleston and Mucke, 1993; Ridet et al., 1997) of molecules which are necessary to control the damage, to diminish its affects and to initiate repair processes.

Although reactive astrocytosis is beneficial to isolate infections and to assist the sealing of damaged blood-brain barrier, it can also be harmful. While glial scarring substantially contributes to the glial cues that inhibit axonal regeneration (Silver and Miller, 2004), reactive astrocytes upregulate synapse-inducing gene expression such as thrombospondins, which facilitates the recovery of CNS insults (Liauw et al., 2008). Another downside of reactive astrogliosis is the possible induction of unwanted synapses that can cause epilepsy or neuropathic pain (Boroujerdi et al., 2008). In addition, mutant astrocytes carrying the sodium dismutase SOD1 (G93A) allele are capable of releasing a toxic signal that can rapidly kill wild-type motor neurons (Di Giorgio et al., 2007; Lobsiger and Cleveland, 2007; Nagai et al., 2007).

Neurological diseases, including demyelinating diseases and epilepsy, can result from mutations in astrocyte-specific genes. More supporting evidence to the major involvement of astrocytes in neuropathologies was reported in various cases. In brain stroke, the lack of blood flow which is followed by reperfusion which has a combined effect. On one side it upregulates antioxidants and free radicals scavengers in the ischemic region and it increases oxidative stress. The astrocytes at the ischemic as well as in the vicinity of ischemic areas play an important role in protecting neurons from mounting oxidative stress (Kraig et al., 1995), and shield the area during the disturbance of blood flow. On the other side, gap junctional communication in astrocytes was reported to propagate and amplify the effects of cell injury by allowing intercellular diffusion of death signals that kill adjacent cells (Lin, 1998). Such a “bystander effect” could account for secondary effects at sites distant from the brain injury.

In an attempt to understand the astrocytes unique contribution in brain pathologies, a few unexpected morphological changes and functional roles of these cells were discovered. For instance, it was shown that in some cases the astrocytes’ domains partially overlap, and they are no longer carefully spaced, but distributed in mix in each other’s territory (Lin, 2008). Furthermore, the connexin (Cx) expression levels changes in response to different cues of specific diseases and this could directly cause shrinking of the astrocytic network. In the case of localized injuries it was observed gradualism and flexibility of the reactive astrocytes response, i.e., Cx expression levels vary in correlation with spatial proximity to the injured place (Ochalski et al., 1995; Theriault et al., 1997; Koulakoff et al., 2008). In brain tissues derived from patients with Alzheimer’s disease (*in situ* analysis), the expression of Cx43 was increased in reactive astrocytes surrounding amyloid plaques (Nagy et al., 1996). After hypoxic preconditioning, Cx43-containing hemichannels release ATP, which is converted to adenosine – a signaling neuromodulator which acting at CNS synapses to restrict synaptic activity and protect neurons (Lin, 2008).

## DNA Damage Response and Ataxia Telangiectasia

An aberrant response to DNA lesions is implicated in many human neurodegenerative disorders (reviewed in Abner and McKinnon, 2004; Barzilai et al., 2008). In healthy cells, the accumulated DNA damage is rapidly detected, leading to the activation of an intricate web of signaling pathways known as the DNA damage response (DDR). On the other hand, in cells with neurodegenerative dysfunction some components of the DDR machinery are impaired (reviewed in Barzilai et al., 2008). Under normal conditions this response culminates in activation of cell-cycle checkpoints and appropriate DNA repair pathways and in certain contexts, initiation of cell death programs. The DDR is a hierarchical process executed through a series of steps. The DNA lesions are detected by sensor proteins that recognize the lesions themselves or chromatin alterations that may result from the DNA damage. Transducers are then brought into action to convey the damage signal to downstream effectors. It is this relay system from transducers to effectors that enables a single DNA lesion to modulate numerous pathways. The transducers might also be involved in the assembly of DNA-repair complexes at the sites of DNA damage (reviewed in Zhou and Elledge, 2000; Iliakis et al., 2003; Shiloh, 2003; Su, 2006). Following double stranded breaks (DSB) induction, the Ataxia Telangiectasia mutated (ATM) protein is activated and a portion of nuclear ATM binds to DSB sites (Andegeko et al., 2001; Meyn et al., 2003). Part of the activation process of ATM involves autophosphorylation of serine 1981 and subsequent dissociation of inactive ATM dimers into active monomers (Bakkenist and Kastan, 2003). The fraction of ATM that binds is also autophosphorylated (Uziel et al., 2003), but recent data indicate that this autophosphorylation is not necessary for ATM recruitment to damage sites (Meyn et al., 2003). Activation of the ATM kinase seems to be an initiating event in cellular responses to irradiation. ATM may be activated by various stresses in addition to DSBs (Kurz and Lees-Miller, 2004). Downstream of the transducer proteins are targets that control various cellular processes such as DNA repair, cell-cycle progression, gene transcription, protein synthesis and degradation, and apoptosis (Banin et al., 1998; Canman et al., 1998; Bakkenist and Kastan, 2003; Lavin, 2007; Matsuoka et al., 2007; Weterings and Chen, 2007; Lempiainen and Halazonetis, 2009; Panier and Durocher, 2009; Bennetzen et al., 2010; Kaidi et al., 2010; Miller et al., 2010; Morris et al., 2010; Nakada et al., 2010; Polo et al., 2010).

## The Chromosomal Instability Syndrome Ataxia Telangiectasia

The ATM protein was identified as the product of the gene that is mutated in the human genetic disease *Ataxia Telangiectasia* (A-T). A-T is characterized by progressive cerebellar degeneration, immunodeficiency, genome instability, premature aging, gonadal dysgenesis, extreme radiosensitivity, and high incidence of lymphoreticular malignancies (for review, see Lavin and Shiloh, 1997; Biton et al., 2008). One of the most devastating symptoms of A-T – cerebellar ataxia – develops progressively into general motor dysfunction. One of the main causes of death of A-T patients is aspiration due to cerebellar-related swallowing defects. Post-mortem studies reveal a significant loss of Purkinje and granule neurons in the cerebellum of children with A-T and therefore once considered as a “Purkinje disease.” Clearly, the cerebellar neurons are seriously damaged due to the loss of ATM, although weather this is a second effect to the glial cells damage, or a parallel independent damage, is yet to be revealed.

According to the glial doctrine, brain pathologies can be to very great extent pathologies of glia which fail to function properly and determine the degree of neuronal death, the outcome and the scale of neurological deficit. If this would apply indeed to genomic instability disorders and A-T in particular, new avenues for treatments could be developed by supplementing the diseased tissue with healthy glial cells and glial-associated supportive factors.

**Figure 1:**
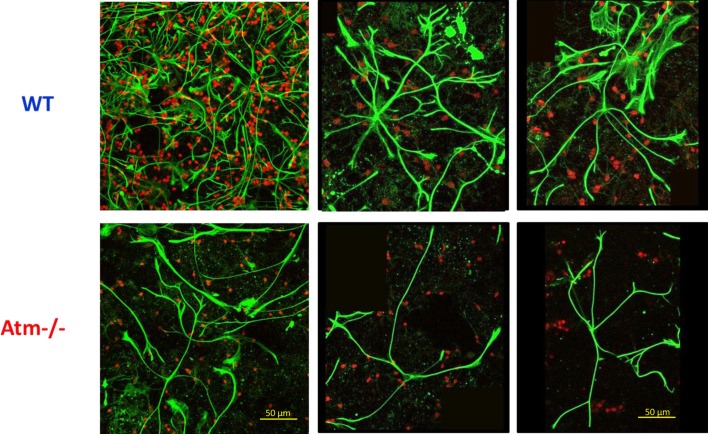
**Glial cell alterations in primary cultures derived from *Atm−/−* mice**. M Immunocytochemical stainingof Glial Fibrillary Acidic Protein (GFAP), a marker of astrocytes (green), and Neuronal Nuclei marker NeuN (red). Note, that *Atm−/−* astrocytes display significantly less processes in comparison to WT astrocytes. Magnification: left column ×20; center and right column ×40.

To test the validity of the glial doctrine we analyzed the role astrocytic morphology in a mouse model of A-T in which the *ATM* gene was knocked out. As shown in Figure 1, the complex arborization of Atm-deficient astrocytes is reduced. In particular, the number of processes originating from a single cell body is significantly lower in *Atm−/−* astrocytes in comparison to the WT cells, with average value respectively of 3.65 ± 1 (*n* = 12; Different representing cells; three different cultures) and 7.5 ± 1.5 (*n* = 12; Different representing cells; three different cultures; *p *< 0.001; Figure 1). To further characterize the glial morphology in other genomic instability disorders we studied the mouse model of Nijmegen breakage syndrome (NBS). NBS is a genomic instability disorder caused by hypomorphic mutations in the Nbs1 gene. When Nbs1 is conditionally inactivated in the central nervous system of mice (Nbs1-CNS-Δ), animals suffer from severe cerebellar atrophy, ataxia, and white matter damage. We observed that conditional inactivation of the murine Nbs1 gene has a profound effect on the integrity and the functionality of the glial cells, which suggests their crucial role in the pathogenesis of NBS (Galron et al., 2011). Conditional disruption of Nbs1 led to a significant reduction and disorganization in the CNS derived from Nbs1-CNS-D mice. Using magnetic resonance imaging (MRI) and region of interest analysis of the T2 maps revealed severe impairment of the white matter in Nbs1-CNS-Δ brains. Biochemical analysis showed low and dispersed staining for myelin basic protein and oligodentrocytes’ progenitor cells in Nbs1-CNS-Δ brains, indicating defects in myelin formation and oligodendrocyte development (Assaf et al., 2008).

**Figure 2:**
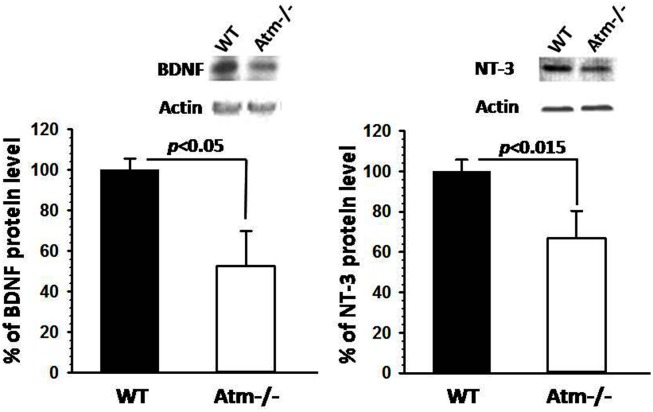
**Reduction in neurotrophic mediators *Atm−/−* mice**. Western blot analysis displaying reduction in BDNF and NT3 (protein levels in *Atm−/−* mice (*n* = 3) and NT3 (*n* = 3). Error bars represent SEM (statistical analysis was performed using two-tailed Students t test.

Additionally, we focused on neurotrophic factors since they play an important role in the maintenance of the CNS homeostasis. Astrocytes are known to produce and secrete their own repertoire of neurotrophic factors, including BDNF and NT3 (Ma et al., 2005). Recent data suggest that a dysfunction in glial cell activity may contribute to the pathogenic process that leads to neurodegenerative Diseases (Ma et al., 2005). Interestingly, Western blot analysis revealed a significant reduction in the levels of BDNF and NT3 in the cerebellum of *Atm−/−* (Figure 2). Collectively, these results suggest that astrocytic cell dysfunction in *Atm−/−* mice play a major role in CNS abnormalities in A-T patients.

## Vasculature

The interactions between astrocytes and endothelial cells are crucial for the formation of the blood–brain barrier (BBB) and in vascular disease pathology. The BBB is a separation of circulating blood from the brain extracellular fluid (BECF) in the CNS. It occurs along all capillaries and consists of tight junctions around the capillaries that do not exist in normal circulation. Endothelial cells restrict the diffusion of microscopic objects (e.g., bacteria) and large or hydrophilic molecules into the cerebrospinal fluid (CSF), while allowing the diffusion of small hydrophobic molecules (O_2_, CO_2_, hormones). Cells of the barrier actively transport metabolic products such as glucose across the barrier with specific proteins. This barrier also includes a thick basement membrane and astrocytic endfeet. The BBB is composed of high-density cells restricting passage of substances from the bloodstream much more than endothelial cells in capillaries elsewhere in the body. Astrocyte cell projections called astrocytic feet (also known as “glia limitans”) surround the endothelial cells of the BBB, providing biochemical support to those cells. The BBB is distinct from the quite similar blood–CSF barrier, which is a function of the choroidal cells of the choroid plexus, and from the blood–retinal barrier, which can be considered a part of the whole realm of such barriers (Hamilton et al., 2007). The interactions between astrocytes and endothelial cells also play an important part in brain development and function. It was demonstrated that in mouse embryos, the endothelin family member Edn3 (Inoue et al., 1989) acting through the endothelin receptor EdnrA (Arai et al., 1990; Sakurai et al., 1990), directs extension of axons of a subset of sympathetic neurons from the superior cervical ganglion to a preferred intermediate target, the external carotid artery, which serves as the gateway to select targets, including the salivary glands (Makita et al., 2008), deliver trophic support as well as differentiation signals to neurons and stem cells (Shen et al., 2004; Dugas et al., 2008), and provide a niche for neural stem cells (Tavazoie et al., 2008). Furthermore, their crucial role at forming BBB is heavily supported in cases of brain injuries (Tavazoie et al., 2008). The reactive astrocytes take critical part in the formation of a shield which encapsulates the brain injury (Bush et al., 1999).

Some brain disorders may have a vascular origin (Forstl et al., 1991; Baldwin and O’brien, 2002), and vascular diseases can be directly linked to neuronal and synaptic dysfunction through changes in the blood flow, increases in BBB permeability, and in changes in nutrient supply (Zlokovic, 2008). As the brain lacks a reserve of glucose and oxygen, it is wholly dependent on a constant blood supply. A threat to cerebral perfusion is likely to have dramatic consequences on neuronal functions. Vascular diseases may lead to activated astrocytes and microglial cells resulting in elevated expression of inducible nitric oxide synthase (iNOS) and release of neurotoxic ROS and nitric oxide. The inflammatory mechanisms may be aggravated by continuous release of chemokines such as CCL2 and CCL3 by astrocytes (McKimmie and Graham, 2010). Furthermore, oxidative stress and glucose starvation may lead to the impairment of astrocyte glutamate uptake, which in turn results in glutamate neurotoxicity. The neurologic symptoms of Aicardi Goutieres syndrome (AGS) are most similar to those patients with Cockayne syndrome (CS). These diseases are characterized by demyelination, brain calcification, and reduced DNA repair. AGS and CS also display vascular degeneration particularly affecting microvessels (reviewed in Brooks et al., 2008). These observations suggest that abnormal vasculature plays a role in the etiology of neurological diseases that are associated with genomic instability.

**Figure 3:**
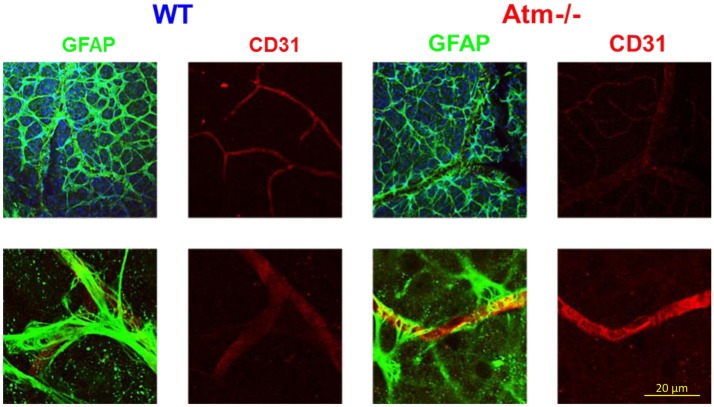
**Glial cell alterations in retinas of *Atm−/−* mice**. Confocal images of flat-mount retinas from WT and *Atm−/−* mice, labeled for CD31 (red) and GFAP (green). Magnification: ×4.

We recently found that *Atm−/−* mice have severely impaired retinal vascularization and leaky blood vessels (Raz-Prag et al., 2011). This finding highlights the neuro-glio-vascular interrelations previously observed (Petzold et al., 2008). We characterized the retina in young and aged Atm-deficient mice. At 2 months of age, angiography revealed slightly impaired retinal vasculature in *Atm−/−* relative to wild-type controls. Labeling for GFAP and CD31 for endothelial cells demonstrated diminution of astrocytes coverage of the vessels in *Atm−/−* retinas (Figure 3). These results suggest that impaired vascularization in the CNS plays an important role in the etiology of A-T and those vascular abnormalities may underlie or aggravate neurodegeneration.

**Figure 4:**
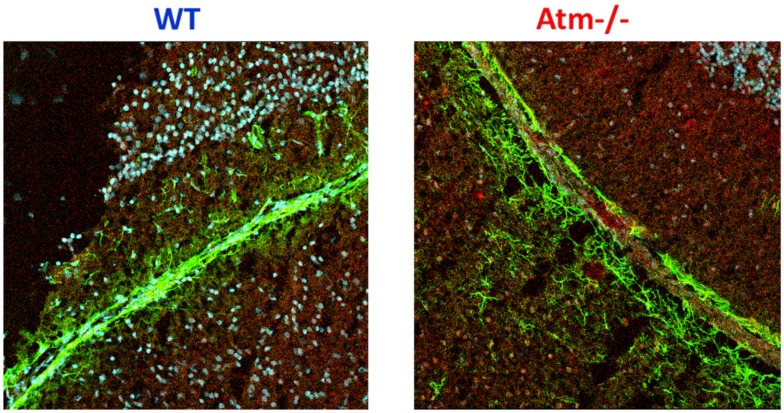
**Alterations in the glial-vascular interactions in cerebellar sections derived from *Atm−/−* mice**. Confocal images of cerebellar sections from WT and *Atm−/−* mice, labeled for CD31 (red) and GFAP (green).

Astrocyte – endothelial cell interaction plays a major role in the function of the vascular unit, and dysfunction in their interaction may lead to microhemorrhages (Lifshitz, 2009). Furthermore, astrocytes are known to regulate the brain’s endothelial barrier by releasing soluble factors such as VEGF (Fruttiger, 2007). In addition to the retinal we investigated cerebellar astrocytes by examining the levels of GFAP in cerebellar slices. Using confocal microscopy, we targeted the interaction of astrocytes (marked by GFAP) with endothelial cells (marked by CD31). As shown in Figure 4, in retinas of WT mice, the processes of the astrocytes appear to form an even network that encases the blood vessels, whereas in WT cerebella the astrocytes fully coat the blood and assume fibrous appearance. On the other hand, in Atm-deficient cerebella, the astrocytes fail to coat the blood vessels and acquired morphology of activated astrocytes, as found in cases of gliosis, and seem to be unable to form a continuous regular net alongside the blood vessels. These results suggest that *Atm* deficiency leads to pathological changes in astrocytes and impairs their ability to support the blood vessels.

**Figure 5:**
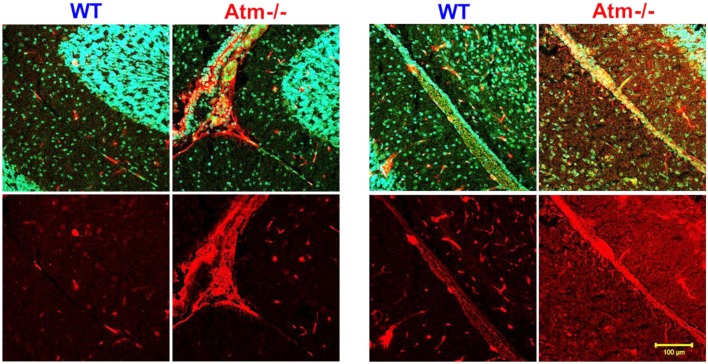
**Increased fibrinogen expression in retinas of *Atm−/−* mice**. Confocal images of cerebellar sections of mice at 2 months of age show markedly increased fibrinogen immunoreactivity (red) in blood vessels (stained with the pan-endothelial marker CD3, green) of *Atm−/−* mice compared to the labeling in WT controls. Cell nuclei are stained with Sytox Blue (blue).

In our previous study (Raz-Prag et al., 2011), we found evidence of vascular leakage in *Atm−/−* retinas. Using confocal microscopy, we found that fibrinogen labeling was observed in clusters outside blood vessels in *Atm−/−* retinas whereas it colocalized with the endothelial cell marker in the retinas of WT mice. Indeed, increases in fibrinogen levels, as well as reduced occludin content, have been previously associated with increased vascular permeability (Antonetti et al., 1998; Tyagi et al., 2008). To further extend this analysis we analyzed vascular integrity in cerebellar slices. Similar to Atm-deficient retinas, Atm deficiency causes increased level of fibrinogen (Figure 5).

**Figure 6:**
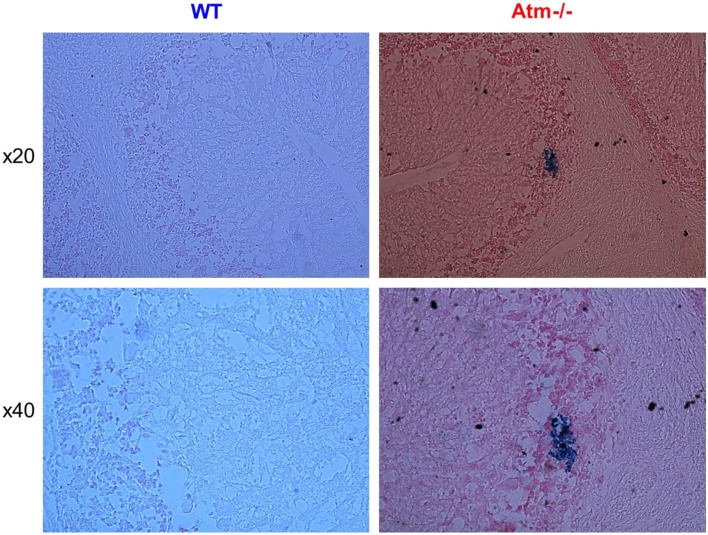
**Vascular leakage in cerebella of *Atm−/−* mice**. Cerebellar section derived from WT and *Atm−/−* mice were stained for hemosiderin to indicate deposits resulting from microhemorrhages. The lower panel shows a larger magnification of WT and *Atm−/−* cerbella. Blue deposits of hemosiderin were evident in cerebella of *Atm−/−* mice (several indicated with arrows), but scarcely in cerebella of WT mice.

To directly demonstrate whether Atm deficiency leads to increased cerebellar blood vessels’ permeability we analyzed the levels of hemosiderin cerebellar slices derived from WT and *Atm−/−* mice. Deposits of hemosiderin are breakdown products of hemoglobin and reflect microhemorrhages that occurred previously (Viswanathan and Chabriat, 2006). Immunohistological analysis revealed deposits of hemosiderin in *Atm−/−* cerebellar slices, whereas WT retinas lacked evidence of such deposits (Figure 6).

## Concluding Remarks

Although a vast body of evidence has documented astrocyte dysfunction, and even dysregulation of astrocyte-specific functions in various neurological diseases, it is still not possible to portray a global unifying picture. This might be, at least in part, due to the fact that the term “astrocyte” covers a heterogeneous group of cells, including highly polarized and differentiated cells as well as stem cells, and all this makes comparison of individual studies difficult (Seifert et al., 2006). Indeed, molecular, functional, and structural definition of astrocyte heterogeneity is a rapidly evolving field, and a better definition of astrocyte subtypes (similarly to what has been provided for GABAergic interneurons in (Ascoli et al., 2008), should greatly promote our understanding of their specific roles in pathophysiology. Here we present evidence that malfunctioning DDR severely affects astrocyte functionality. We found that Atm deficiency significantly reduced processes’ complexity of astrocytes. We also discovered a significant reduction in the secretion of neurotrophic factors, such as brain-derived neurotrophic factor and neurotrophin 3. Furthermore, our data suggest an important role for astrocyte–vascular interaction in Atm-deficient mice and probably A-T patients. Atm deficiency leads to increased blood vessels’ permeability evidenced by increased fibrinogen levels and microhemorrhages.

Another important open question is whether astrocytic networks affect neuronal activity (reviewed in Giaume et al., 2010). So far, this question has been rather difficult to address owing to the lack of specific tools for studying astrocyte Cx channels.

Mathematical modeling of neuroglial interactions could help to determine the role of single astrocytes and astrocytic networks. While several groups have attempted to create a coherent model of astrocyte calcium signal propagation and responses to neuronal activity, only a few have modeled the feedback of astrocytic activity on neurons (De Pittà et al., 2011). The use of computer simulations trying to imitate those processes could yield significant advances in understanding the neuroglial interactions.

Progress in the field of glio-biology during the past few years indicates that this could lead to the development of novel cell-centered therapeutic approaches to neurological disorders.

## Conflict of Interest Statement

The authors declare that the research was conducted in the absence of any commercial or financial relationships that could be construed as a potential conflict of interest.
